# Thrombus or vegetation?Importance of cardiac MRI as a diagnostic tool based on case report and literature review

**DOI:** 10.1016/j.amsu.2020.12.007

**Published:** 2020-12-05

**Authors:** Jamal El ouazzani, Issam Jandou, Issam Christophe Thuaire

**Affiliations:** a Department of Cardiology, Mohammed VI University Hospital Center, BP 4806 Oujda Université, 60049, Oujda, Morocco; b Department of Urology, Ibn-Rochd University Hospital Center, 20340, Casablanca, Morocco; c Department of Cardiology, Hôpital Louis Pasteur, Chartres, France

**Keywords:** Late gadolinium enhancement magnetic resonance imaging, Thrombus, Vegetation, Case report

## Abstract

**Introduction:**

We report the Case of a 35 years old male patient admitted for pulmonary embolism in a febrile context. Transthoracic echocardiography showed a filamentary mass appended to the pulmonary valve whose thrombotic origin has been suggested on data of late gadolinium enhancement magnetic resonance imaging.

**Case presentation:**

The patient had a history of deep vein thrombosis in the context of familial thrombophilia with factor V leiden gene mutation in two of his sisters and an inhaled drug addiction to heroïn. There was a biological inflammatory syndrome with negative blood cultures. Transthoracic echocardiography showed a very mobile homogeneous hyperechoic mass measuring 8 cm in the right ventricle appended between the pulmonary valve and the lateral wall of the RV. In LGE-MRI, an isointense, to the myocardium, marginal hall and a central rim enhancement were objectified, suggesting the diagnosis of thrombus rather than vegetation.

**Conclusion:**

Despite the notion of drug addiction, the febrile context and the localization of the mass, a diagnosis of RV thrombus rather than infective endocarditis was favored relying on familial thrompbophilia, personal history of DVT and LGE-MRI aspect. The patient was treated with curative heparin therapy and antibiotic therapy. Due to the persistence of the mass after three weeks of treatment and after heart-team discussion, the patient underwent surgical mass removal. The anatomopathological study confirmed a fibrino-cruoric thrombus.

## Highlights

• Differential diagnosis between thrombus and vegetation is sometimes a real challenge for the clinician.
• Cardiac MRI with its LGE-MRI sequences can help characterizing thrombus with hypointense edges and brighter central zone called “the etched appearance”.
• Even in the multimodality imaging era, anatomopathological stady sometimes is needed to reach the correct diagnosis.

## 1 Introduction

The diagnosis of infective endocarditis is made based on the modified Duke's criteria, including clinical presentation, results of blood tests, blood culture and imaging, including echocardiography and PET Scan [1]. Echocardiography is recommended as first-line diagnostic imaging and has high diagnostic performance. However, its ability to distinguish between vegetation, thrombi and cardiac tumor is limited [1].

Computed tomography and MRI technical developments leading to high-quality images and thorough information in this matter [2,3]. Multimodality imaging play an increasing role in differential diagnosis of these three types of heart masses.

However, very few studies have reported results on the diagnostic value of cardiac MRI to discriminate thrombus from vegetation and no characteristic MRI signs of vegetation have been reported.

We report a Case of a serpentine mass measuring 10 cm appended to the pulmonary valve with a challenging diagnostic between thrombus and right heart endocarditis. We will discuss the contribution of cardiac MRI and especially late gadolinium enhancement sequences in this diagnostic approach. **The work has been reported in line with the SCARE 2018 criteri**a [4].

## 2 Presentation of case

We report the Case of a 35-year-old male patient, chronic smoker, with a history of inhaled heroin addiction and DVT treated with Rivaroxaban for 6 months, and there is a family history of Factor V Leiden in two siblings. The clinical examination showed a preserved venous capital with absence of dermatological signs pointing towards intravenous drug injection.

**Fig. 1:**
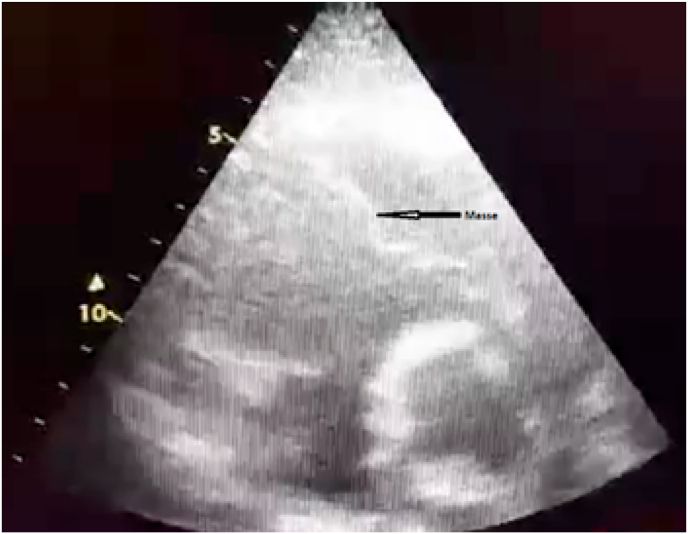
TTE, parasternal long axis view centered on the right chambers of the heart showing the filamentary mass appended between the pulmonary valve and the lateral wall of the right ventricle.

The patient presented to the emergency room with dyspnea at rest, associated with chest pain of the right hemithorax accentuated with deep inspiration. Upon presentation, vitals were as follows: oral temperature of 38.7∘ C, blood pressure of 130/90 mmhg, heart rate of 80 beats/min, and respiratory rate of 22 breaths/min, with an oxygen saturation of 88% on room air. The patient was alert and oriented. ECG showed sinus rhythm with a heart rate at 80 bpm. The thoracic CT angiography showed a pulmonary embolism of the right intermediate trunk with images of alveolar condensations which could be related to pulmonary infarctions or septic localizations. TTE revealed a mobile, serpentine, hyperechoic mass, appended between the lateral wall of the right ventricle and the pulmonary valve measuring 8 cm (Fig. 1). In addition, the pulmonary valve was intact without regurgitation or obstruction of the pulmonary outflow tract obstruction. The trunk of the pulmonary artery was free. The RV was of normal size and function. The left ventricle was of good function. Laboratory tests revealed elevated levels of CRP of 148 mg/l, leukocytosis of 13,000/mm3. NT-proBNP and troponin were normal. The blood cultures were all negative. The cerebral and thoraco-abdominopelvic CT scan, performed as part of the extension assessment in the hypothesis of an IE were normal.

In this context of diagnostic doubt between an IE and thrombus, the patient was treated by a curative anticoagulation based on enoxaparin sodium in association with an intravenous antibiotic therapy: vancomycin and gentamycin for one week then Rifampicin and ciprofloxacin because of fever and inflammatory syndrome persistence. Tests of classic causes of blood culture negative endocarditis were negative.

Close monitoring of the patient showed improvement with regression of dyspnea, chest pain, fever and inflammatory syndrome after three weeks of antibiotic and anticoagulant therapy, but persistence of the mass with no decrease.

**Fig. 2:**
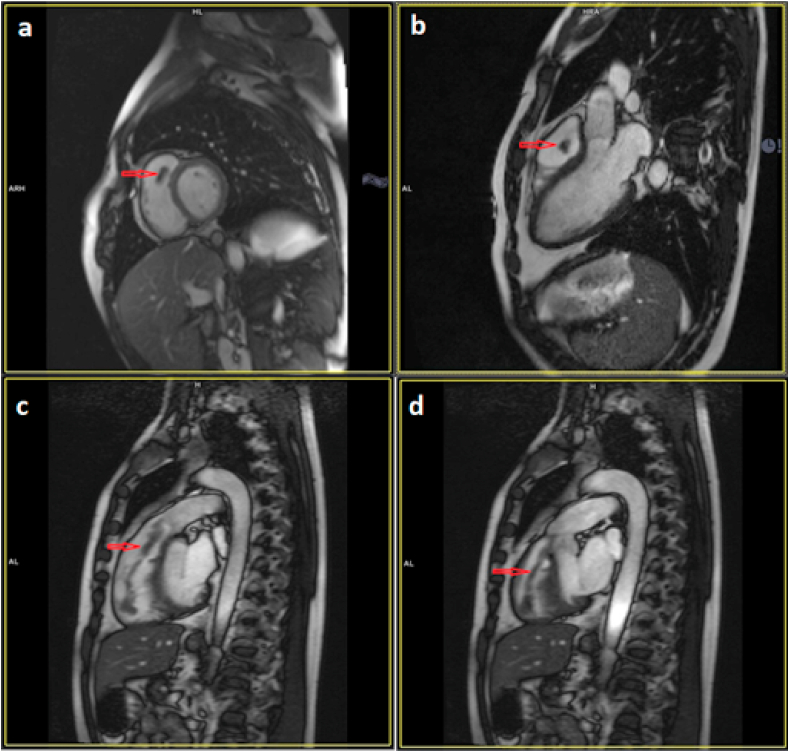
Cardiac MRI performed 3 weeks after patient admission, showing an iso-signal intensity (compared with normal myocardium) filamentary mass on cine SSFP (steady state free precession) sequences. (a) short axis view, (b) three chamber view, (c, d) sagittal view through the right ventricle and the pulmonary trunk. Siemens Avanto 1.5T Siemens Healthineers.

**Fig. 3:**
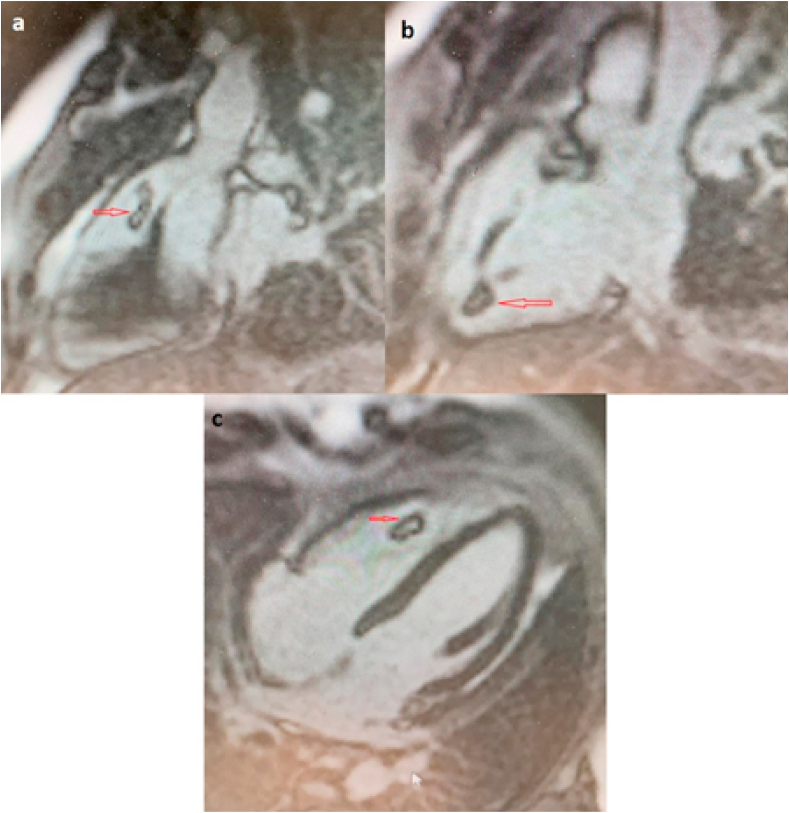
LGE-MRI, PSIR-type sequences, showing an «“etched” appearance » of the mass (hypointense border and brighter central zone). Siemens Avanto 1.5T Siemens Healthineers.

A cardiac MRI was performed, it showed a serpentine mass of 10 cm, appended between the pulmonary valve and the RV apex causing a partial obstruction of right ventricular outflow tract. This mass had a signal intensity on the SSFP cine MRI imaging sequences identical to that of the myocardium (Fig. 2, a-d). On the LGE-MRI sequences, in particular on the PSIR sequences, there was an “etched appearance” with a hypointense border and a central brighter zone (Fig. 3), favoring the diagnosis of an organized thrombus.

Given the unchanged dimensions of the mass despite antibiotic and anticoagulant treatment, the potential risk of its migration in the pulmonary trunk and after heart team discussion, it was decided to surgically remove the mass.

The surgical procedure, **made by experienced team of heart valve surgery from Paris,** allowed to complete resection of the mass which was 10 cm long with preservation of the healthy native pulmonary valve. Anatomopathological analysis confirmed the purely thrombotic nature of the mass.

## 3 Discussion

The diagnosis of infectious endocarditis is based on clinical and biological data, including blood culture, echocardiography and 18F-FDG PET/CT. This diagnosis remains difficult due to the atypical clinical manifestations and the low specificity of the Laboratory tests [1]. Right-sided infective endocarditis is less common than left-sided, it is more frequent in intravenous drug users or patients with underlying congenital heart disease corrected or not [2,3].

Echocardiography, either TTE or TEE, is the first imaging technique used for diagnosis purpose, it shows high sensitivity and specificity, especially when performed in a complementary manner. TTE/EET have limited diagnostic performance in differentiating thrombi from vegetations or cardiac tumors, especially at the early stage and when the vegetation is too small (2–3 mm) to be detected or when the mass is appended to a valve. In our Case, TTE and TEE showed a very mobile serpentine mass measuring 8 cm in length appended to the pulmonary valve. But this echocardiographic aspect did not allow to differentiate thrombus or vegetation. The patient was febrile; but blood cultures and tests for classic causes of blood culture negative endocarditis were negative.

The role of MSCT and CMR imaging for the diagnosis of infectious endocarditis has been poorly evaluated [1,5,6]. Vegetation cardiac MRI aspects have not been clearly depicted in literature. Differentiation between vegetation and thrombus can be difficult, because both can have a similar aspect on LGE-MRI [7]. In Case of infective endocarditis, some authors report cases of marginal mass enhancements on LGE-MRI [8], which could facilitate the differentiation between vegetation and thrombus. Other results suggest that cardiac MRI may show endocardial LGE reflecting irreversible endocardial damage or fibrosis, and may also show perivalvular abscess [[9], [10], [11]]. Dursun and al, have shown that even in the absence of vegetation detected on cardiac MRI or echocardiography, the detection of late endocardial enhancement indicating endothelial inflammation can contribute to the diagnosis of infective endocarditis and guide therapeutic management [9]. Zatorska K and al, have shown that cardiac MRI can be useful in diagnosing perivalvular complications in IE patients, although vegetation visualization is limited by the low spatial resolution of this method. In this study LGE associated with inflammatory process extension and myocardium infiltration was reported in 40% of patients [12].

In Case of thrombus, signal intensity is changing depending on the thrombus age and it does not generally enhance in LGE-MRI sequences. Cardiac MRI late enhancement sequences can detect thrombus with higher sensitivity than echocardiography by describing a high contrast between the dark thrombus and the adjacent structures.

Mural thrombus does not enhance during the first-pass perfusion (FPP) and is often depicted with hypointense border and brighter central zone (“etched appearance”) particularly on PSIR-type sequences, allowing high diagnostic specificity [13].

One pattern was found to be more common in thrombi (94%) than in tumor (2%) in a study: hyperintensity at short inversion time (TI), “edged” appearance with hypointense border and brighter central zone at intermediate TI, and hypointensity pattern at long TI. This pattern had the highest accuracy (95%) to differentiate thrombus and tumor [14]. The same study showed that combination of negative FPP, negative LGE and typical thrombus pattern in TI scout sequence had a specificity of 98% for thrombus, whereas the combination between negative FPP and negative LGE had a specificity of 92% for the diagnosis of thrombus [14].

Few cases have been reported describing cardiac masses with T1 and T2 relaxation time measurement. Saba et al. reported that the mean T1 relaxation time of the cardiac thrombus and myxoma were 1044.7 ms and 1681.6 ms, respectively [15].

The cardiac ECG synchronized computed tomography provides high resolution anatomical information in cases of infective endocarditis, especially in Case of valve prosthesis. It can highlight vegetation, aneurysm or perivalvular abscess.

18F-FDG PET/CT imaging may also, particularly in patients with prosthetic valves, be useful in the IE diagnosis showing abnormal 18F-FDG uptake around prosthetic valve IE with high sensitivity [1,6].

**Conclusion:** Differential diagnosis between thrombus and vegetation is sometimes a real challenge for the clinician. Cardiac MRI with its LGE-MRI sequences can help characterizing thrombus with hypointense edges and brighter central zone called “the etched appearance”.

## Consent

Written informed consent was obtained from the patient for publication of this case report and accompanying images. A copy of the written consent is available for review by the Editor-inChief of this journal on request.

## Funding

This Case report did not receive any specific grant from any funding agency in the public, commercial or not-for-profit sector.

## Author contribution

Dr. IJ, and Dr. CT analysed and performed the literature research, Dr. EJ performed the examination and performed the scientific validation of the manuscript. Dr. El ouazzani Jamal was the major contributors to the writing of the manuscript. All authors read and approved the manuscript.

## Consent

A copy of the written consent is available for review by the Editor-in-Chief of this journal on request”.

## Provenance and peer review

Not commissioned, externally peer-reviewed.

## Registration of research studies

1. Name of the registry:
2. Unique Identifying number or registration ID:
3. Hyperlink to your specific registration (must be publicly accessible and will be checked):

## Ethical approval

Research studies involving patients require ethical approval. Please state whether approval has been given, name the relevant ethics committee and the state the reference number for their judgement.

## Guarantor

Dr. Jamal El ouazzani.

## Declaration of competing interest

All authors have non-financial conflicts of interest related to this report.
